# Extracellular-Matrix Mechanics Regulate the Ocular Physiological and Pathological Activities

**DOI:** 10.1155/2023/7626920

**Published:** 2023-07-22

**Authors:** Ran Zhang, Bo Li, Heng Li

**Affiliations:** ^1^Department of Ophthalmology & Optometry, North Sichuan Medical College, Nanchong 637000, Sichuan, China; ^2^Department of Ophthalmology, Central Hospital of Suining City, Suining 629000, Sichuan, China

## Abstract

The extracellular matrix (ECM) is a noncellular structure that plays an indispensable role in a series of cell life activities. Accumulating studies have demonstrated that ECM stiffness, a type of mechanical forces, exerts a pivotal influence on regulating organogenesis, tissue homeostasis, and the occurrence and development of miscellaneous diseases. Nevertheless, the role of ECM stiffness in ophthalmology is rarely discussed. In this review, we focus on describing the important role of ECM stiffness and its composition in multiple ocular structures (including cornea, retina, optic nerve, trabecular reticulum, and vitreous) from a new perspective. The abnormal changes in ECM can trigger physiological and pathological activities of the eye, suggesting that compared with different biochemical factors, the transmission and transduction of force signals triggered by mechanical cues such as ECM stiffness are also universal in different ocular cells. We expect that targeting ECM as a therapeutic approach or designing advanced ECM-based technologies will have a broader application prospect in ophthalmology.

## 1. Introduction

A large number of studies are increasingly concerned with the physical properties of cells and tissues along with the influences of biophysical characteristics within tissue microenvironments on cell function [1–3]. There is growing evidence that external mechanical factors impact the configuration of biomacromolecules, the fate of cells, and the structure of tissues [4, 5]. The extracellular matrix (ECM) is a common scaffold for maintaining the homeostasis of tissues and organs [6]. It forms a complex but highly organized network and is remodeled dynamically around cells that not only provides mechanical support for the completeness and resilience of cells but also modulates cell homeostasis and signal transduction [7–9]. ECM is a crucial component of the ocular microenvironment, which plays an essential role in every part of the eye, either maintaining the transparency and hydration of the cornea and vitreous, or modulating angiogenesis, intraocular pressure (IOP) maintenance, and vascular signaling [10, 11]. ECM is the noncellular structure composed of water, collagens, and glycoproteins, and all tissues have the ECM with an individual composition and topology, which is developed through the crosstalk and interplays of biochemical and biophysical between manifold cellular components (such as epithelial, fibroblast, adipocyte, endothelial elements) as well as a constantly remodeled cellular and protein microenvironment during tissue development [12–14].

ECM stiffness, a source of mechanical stimulation, transfers the external physical force onto the cell, which is transformed into biochemical signals through a series of cascade reactions inside the cells to regulate various ocular physiological and pathological activities, including cell migration and proliferation, retinal vascular development, corneal homeostasis, maintenance of normal human trabecular mesh function, and IOP as well as the occurrence of glaucoma and subconjunctival fibrosis [12, 15–17].

In this review, we summarize the major components and physical properties of ECM and its underlying mechanisms for regulating normal ocular physiological and pathological activities. We aim to provide new insights into how the multiple signals are integrated to modulate ocular cell behaviors for the development of novel therapeutic strategies for ocular tissue repair and regeneration while targeting ECM as a therapeutic modality.

## 2. ECM Stiffness in the Cornea

The cornea is a transparent tissue located on the anterior surface of the eyeball and is the eye's major refractive structure, providing most of the refractive power needed to focus light onto the retina [18]. The cornea is composed of the outer epithelium, the stroma, and the inner endothelium, of which the stroma accounts for more than 90% of the corneal thickness [19]. The corneal stroma is an ECM with enrichment of collagen and highly ordered characteristics, which provides clarity and preserves the structure necessary for light refraction after assembly [18, 20]. In addition, the abundance of collagen I in the ECM confers the cornea in biomechanical stability and form [21, 22]. Corneal keratocytes are stroma-resident cells in charge of maintaining the ECM's highly organized structure and the homeostasis of its constituent parts [21, 23]. After surgery or trauma, the mechanical characteristics of the cornea will change significantly during wound healing, leading to a substantial increase in ECM stiffness [24–26]. This is due to the fibrotic reaction caused by the activation of corneal cells from a natural mechanically quiescent condition to the active myofibroblast state, which distorts the highly ordered structure of the ECM, resulting in corneal turbidity and even visual impairment [15]. Since it has been shown that transforming growth factor-*β*1 (TGF-*β*1) is released into the stromal space after injury and can promote the differentiation of dormant keratocytes into myofibroblasts, the phenotypic transition of corneal keratocytes is linked to signaling downstream of TGF-*β*1 [27–29]. Primary corneal keratocytes were cultured on collagen-coated glass coverslips or a stiff (10 kPa) gel matrix, which resulted in mechanical dependence with a wider variety of morphology, plentiful stress fiber formation, higher levels of *α*-smooth muscle actin (*α*-SMA) expression, and stronger traction forces [15, 30]. Conversely, corneal cells cultivated on a soft (1 kPa) gel produced fewer stress fibers and kept more of their dendritic morphology, indicating a dormant keratocyte phenotype [15]. These results emphasize the crucial role that ECM stiffness plays in controlling the mechanical phenotype of corneal cells during corneal wound healing.

The biomechanical properties of the cornea, including ECM stiffness, have recently been used in clinical risk assessments to diagnose glaucoma and predict disease progression [31, 32]. Corneal biomechanics change with age and loss of stromal tissue was observed in corneal pathology [33, 34]. The three key proteins lysyl oxidase (LOX), transglutaminase-2 (TGM-2), and advanced glycation end products (AGEs) are responsible for more collagen crosslinking, which causes an excessive increase in ECM stiffness and causes ocular stiffness in glaucoma, reflecting the significant role ECM plays in the mechanical homeostasis of the eye [31, 35, 36].

## 3. ECM Stiffness in the Retina

Mammalian vision begins with the transmission of light through the cornea and lens to the retina, which is the layer of cells along the rear wall inside the eye [37]. The extremely professional retina finishes the conversion of energy from absorbed photons into neural activities, and the brain consequently can elucidate the patterns of detected photons [37, 38]. ECM molecules are found mostly in the basement membrane (BM) and nonbasement membrane (NBM) of the retina [39]. BM is linked to three types of retinal tissue: Bruch's membrane, inner limiting membrane (ILM), and vasculature, and their major physiological role is to demarcate the neural retina from surrounding non-neural tissue [16, 40, 41]. Typically, the NBM is usually located between the retinal pigment epithelium (RPE) and the ILM [16].

**Figure 1:**
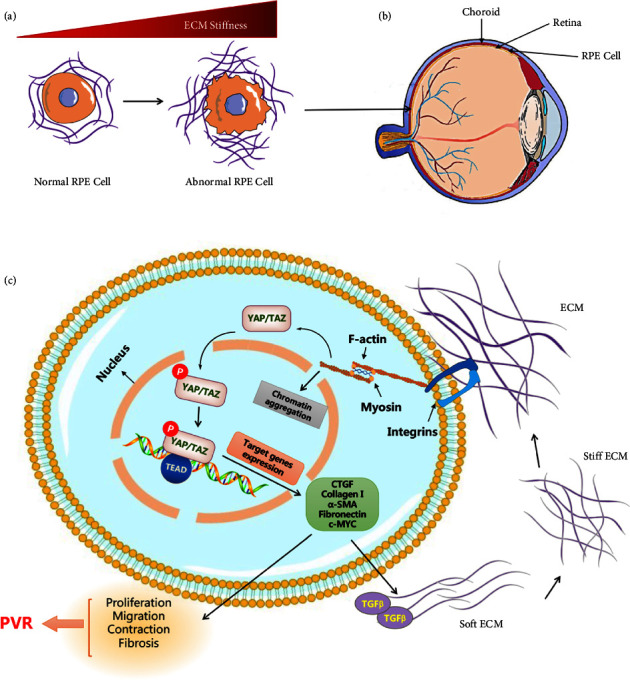
ECM stiffness-regulated mechanical signaling pathways in RPE cells. (a) Increased ECM stiffness leads to the generation of abnormal RPE cells. (b) The RPE cells are located outside the retina and attached to the choroid. (c) Increased ECM stiffness modulates intracellular downstream signaling molecules such as YAP/TAZ and F-actin through integrins and other transmembrane proteins, thereby promoting phosphorylation of YAP and its subsequent translocation of nuclear where it interacts with the TEAD family of transcription factors to form transcription complexes that further regulate RPE cell migration, proliferation, and contraction of target genes (including CTGF, collagen I, *α*-SMA, c-MYC) that are implicated in PVR development. RPE: retinal pigment epithelium. PVR: proliferative vitreoretinopathy. CTGF: connective tissue growth factor. *α*-SMA: *α*-smooth muscle actin. TGF-*β*: transforming growth factor-*β*.

In the retina, ECMs are widely distributed throughout the nerve fiber layer, the outer and inner plexiform layers, and the interphotoreceptor matrix [41]. Muller glial cells, intraretinal glial cells as well as migrating astrocytes represent the major sources of ECM secretion [16, 42, 43]. ECM creates the environment around retinal cells, serves as the BMs, and offers structural and mechanical support. Meanwhile, its constituents are essential for the differentiation and development of the retina [16, 44]. The development of retinal microvessels is regulated by the laminin family, and alterations in the collagen, elastin, and tenascin-C content of the ECM will increase axonal injury [45]. Following ischemia, neurodegeneration of the retina and optic nerve is associated with the remodeling of several ECM molecules [44]. The elastic modulus of retinal cells ranges from 200 Pa to 1000 Pa, and a variety of pathological reactions in the retina lead to local variations in retinal tissue stiffness [46]. The considerable changes in retinal rigidity brought on by age, laser surgery, and retinal detachment are mostly attributed to the ECM [47]. Excess ECM deposition leads to increased ECM stiffness, inducing the activation of RPE cells together with the associated complement system, which in turn promotes the epithelial-mesenchymal transition (EMT) of RPE cells to initiate subretinal fibrosis (Figures 1(a) and 1(b)) [48, 49]. Strikingly, the underlying mechanism is that ECM stiffness regulates RhoA GTPase activity and the actin cytoskeleton to activate mechanically sensitive molecules Yes-associated protein/transcriptional coactivator with pdz-binding motif (YAP/TAZ) and then drives their translocation into the nucleus, where they interact with the TEAD family of transcription factors to form the complex (Figure 1(c)) [47]. Intracellular distribution and expression of YAP are regulated by ECM-related mechanical stiffness, which is associated with pathological fibrosis, and overexpression of YAP has been detected in renal, lung, and liver fibrosis [47]. The transcription complex then regulates RPE cell migration, proliferation, and contraction through target genes that are implicated with the development of proliferative vitreoretinopathy (PVR) (Figure 1(c)) [47].

*In vitro* experiments found that compared to cells cultivated on the soft gel (0.5 kPa) matrix, cultured ARPE-19 cells (human retinal pigment epithelial cell line) on rigid (50 kPa) gel coverslips showed significantly increased ECM production and nuclear translocation of YAP as well as significantly elevated protein levels of its downstream targets, connective tissue growth factor (CTGF), and CYR61, which are the signaling molecules associated with retinal fibrosis (Figure 1(c)) [47, 50]. In addition, it was shown that inhibiting YAP and RhoA reduced the levels of tumor necrosis factor (TNF)-*α*, interleukin (IL)-1*β*, and IL-6 induced by ECM stiffness in the retina. These results suggest that targeting ECM or blocking YAP and RhoA signaling pathways can reduce PVR-induced retinal fibrosis and alleviate persistent retinal inflammation *in vivo* (Figure 1(c)) [47].

## 4. ECM Stiffness in the Trabecular Meshwork

**Figure 2:**
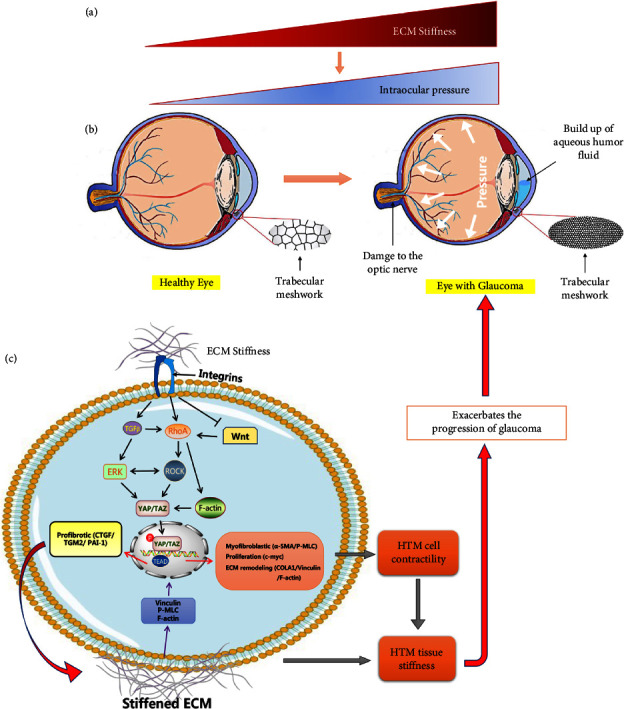
Schematic effects induced by ECM stiffness and TGF*β* regulating YAP/TAZ activity in HTM cells. (a) The increase in ECM stiffness promotes elevated intraocular pressure. (b) Ocular pathologic features of the eye with glaucoma compared to the normal eye. (c) Upregulation of ECM stiffness may regulate the formation of focal adhesion and actin skeleton rearrangement to improve YAP/TAZ activity and its nuclear translocation. Meanwhile, ECM stiffness activated TGF*β* triggers ERK and ROCK signaling pathways, which also promotes the upregulation of YAP/TAZ activity. The transcription complex formed by YAP/TAZ and TEAD in the nucleus activates a variety of mechanically stimulated target genes (TGM2, FN, CRGF, PAI-1, vinculin, *α*-SMA, P-MLC, F-actin) to promote cell contractility and ECM remodeling in HTM, which can aggravate the tissue stiffness of HTM, lead to fibrotic phenotype, enhanced aqueous humor outflow resistance, increased IOP, and exacerbate the progression of glaucoma. In addition, the inhibition of the Wnt signaling pathway can promote RhoA to initiate downstream ECM stiffness-raising phenotype, finally leading to elevated IOP. CTGF: connective tissue growth factor. *α*-SMA: *α*-smooth muscle actin. COLA1: alpha-1 type I collagen. c-myc: the regulator genes and proto-oncogenes that code for transcription factors. TGM2: transglutaminase 2. P-MLC: phospho-myosin light chain. PAI-1: plasminogen activator inhibitor-1. F-actin: actin filaments. Vinculin: a membrane-cytoskeletal protein in focal adhesion plaques.

Trabecular meshwork (TM) provides the majority of the flow resistance to the outflow of aqueous humor, and the manipulation of trabecular outflow resistance is responsible for the modulation of IOP [51, 52]. ECM is an important constituent of all parts of the TM: the corneoscleral, uveoscleral, and juxtacanalicular layers [10, 53]. ECMs in TM consist of glycosaminoglycans and proteoglycans, collagens, elastic fibrils, basement membrane, and matrix proteins, and they determine the TM stiffness [51, 54]. The outflow channel of ECM is quite dynamic and undergoes continuous turnover and remodeling, which is contributing to regulating the homeostasis of IOP (Figures 2(a) and 2(b)) [55, 56]. Environmental stimulation, such as mechanical stretch or a series of growth factors, cytokines, and drugs (such as dexamethasone), will alter the expression of ECM components and its mechanical properties, triggering the continuous increase of trabecular outflow resistance, leading to the enhanced IOP, which is the main risk factor for glaucomatous optic neuropathy and a link to deteriorating vision [51, 57, 58].

The progression of primary open-angle glaucoma (POAG) was associated with the increased stiffness of human TM (HTM) and enhanced levels of transforming growth factor-*β*2 (TGF-*β*2) in aqueous humor [59]. ECM stiffness has been shown to profoundly alter the cytoskeletal structure and kinetics of HTM [60, 61]. Through the biomimetic ECM hydrogels or polyacrylamide substrates with tunable stiffness to explore the mechanism of ECM regulation of glaucoma, substrate stiffness significantly enhanced TM cell spread and altered TM cell morphology [59, 62]. Multi-omics analysis and immunostaining revealed that prominent stress fibers observed on stiff substrates were related to the formation of focal adhesion and that a distinct stiffness-induced reorganization of the actin cytoskeleton [59, 63]. Stiffer substrates also promoted increased TM cell proliferation. Moreover, increased substrate stiffness was found to alter the intrinsic TM cell stiffness profile (Figures 2(a)–2(c)) [64]. More importantly, stiffer ECM hydrogels up-regulated TGF-*β*2 expression and YAP/TAZ activity as well as their translocation in the nucleus [59]. TGF-*β*2 has been shown to induce nuclear YAP/TAZ localization and the consequent activation of target genes (which are correlated with the development of glaucoma), including TGM-2, CTGF, and plasminogen activator inhibitor-1 (PAI-1), via ERK and ROCK signaling pathways in response to mechanical signals (Figure 2(c)) [59, 65, 66]. Of note, the Wnt signaling avenue, another vital mechanism in glaucoma, is involved in regulating ECM stiffness in response to mechanical signals influenced by YAP/TAZ (Figure 2(c)) [67]. The antagonistic effects of Wnt signaling are a cause of elevated IOP, or the expression of component Wnt is crucial for preserving normal IOP in HTM (Figure 2(c)) [66]. These results confirm the impact of ECM stiffness on TM cells, and elevated stiffness may contribute to the fibrotic behavior of TM cells in glaucoma, suggesting that therapies targeting ECM or ECM-related mechanical signaling molecules can be developed to prevent the occurrence and development of glaucoma.

## 5. ECM Stiffness in the Optical Nerve

ECM is constantly remodeled in the optic nerve, and its various components, including glycoproteins fibronectin, laminin, tenascin-C, and tenascin-R as well as the chondroitin sulfate proteoglycans (CSPGs), aggrecan, neurocan, and brevican, are critical for maintaining the normal function of the optic nerve [68–70]. Tenascin-R can modulate neurite outgrowth as well as neural and glial adhesion, whereas tenascin-C is increased and engaged in neuroinflammation and glial response under pathological conditions, and CSPG is highly aggregated in glial scars, limiting axon's ability to regenerate [44, 71]. Axon damage can be exacerbated by changes in ECM composition, and remodeling of several ECM components is linked to neurodegeneration of the retina and optic nerve following ischemia [16, 71]. The retinal ganglion cell (RGC) axons' egress as well as the entrance and exit of the retinal blood vessels are made possible by the optic nerve head (ONH), a structure in the posterior ocular fundus. Collagenous load-bearing beams make up the lamina cribrosa (LC), a porous support system that spans the ONH and provides protection for fragile, unmyelinated RGC axons as they depart the eye posteriorly to form the optic nerve [72, 73]. LC is made up of ONH astrocytes and fibroblast-like cells (referred to as LC cells), which are essential for maintaining the surrounding ECM [72, 74].

**Figure 3:**
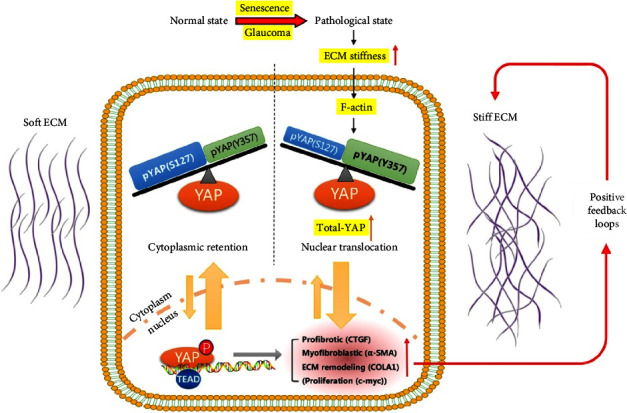
Regulation of ECM stiffness in normal and pathological LC cells. Regulation of ECM stiffness in healthy and diseased LC cells. The diagram of a healthy LC cell in soft ECM (left) transforming into a pathological state (right) in a stiffer matrix. When ECM stiffness increases with aging or glaucoma, F-actin increases followed by YAP phosphorylation at tyrosine 357 elevates, while its serine 127 phosphorylation decreases, but the total YAP augments, resulting in elevated nuclear translocation and enhanced nuclear import (to some extent through direct F-actin-mediated pore opening) as well as lowered nuclear export, which correspondingly promotes the transcriptional activity of targets, leading to upregulation of ECM protein expression, enhanced myofibroblastic markers, incremental activation growth factors, and increased proliferation [72]. A “positive feedback loop” on the right illustrates how this cycle is self-sustaining. CTGF: connective tissue growth factor. *α*-SMA: *α*-smooth muscle actin. COLA1: *α*-1 type I collagen. c-myc: the regulator genes and proto-oncogenes that code for transcription factors.

Significantly larger cell spread area and enhanced actin filament growth and the creation of more vinculin-focal adhesions (number and size) were observed in both normal and glaucoma LC cells cultured on stiff silicon elastomer surfaces (100 kPa) [74]. These alterations were shown to be positively correlated with enhanced cell stiffness as evaluated by atomic force microscopy (AFM) [75, 76]. Proliferation and cytoskeletal alterations in glaucoma LC cells were noticeably greater than in normal cells. Both LC cells exposed to a stiffer substrate underwent the change into a myofibroblast-like phenotype, as shown by enhanced *α*-SMA signaling and its colocalization with actin stress fibers [74]. Interestingly, normal LC cells cultured on a mimicked stiffness substrate (100 kPa) showed significantly up-regulated YAP gene and protein expression, followed by elevated YAP phosphorylation at tyrosine 357, and decreased YAP phosphorylation at serine 127 (Figure 3). This differential phosphorylation expression results in an enhancement in total-YAP with an increase in nuclear translocation and aggregation as well as a decrease in nuclear export, thus promoting the transcriptional activity of YAP/TEAD complex transcription targets, which leads to elevated ECM protein synthesis, enhanced myofibroblast markers, augmented activation of connective tissue growth factors, and increased proliferation, eventually forming a feedforward cycle mode (Figure 3) [72]. Notably, treatment with the YAP-specific inhibitor verteporfin remarkably disrupted mechanotransduction in LC cells triggered by enhanced ECM stiffness, eliminating any subsequent pro-fibrosis processes and positive feedback loops [72].

These results showed that a stiffer cell microenvironment stimulates a myofibroblastic transition in human LC cells, thus contributing to LC remodeling and fibrosis in glaucoma.

## 6. The Role of ECM in the Vitreous Body

The human vitreous body is a nearly spherical transparent structure, about 4.5 milliliters in volume, which is encircled by and attached to the retina, pars plana, and lens of the eye [77]. The vitreous body is a highly hydrated, almost acellular, transparent tissue with various ECM components, including thin heterotypic collagen fibers composed of type II, type IX, and type V/XI collagen that are crucial to the gel structure [78, 79]. Hyaluronan is the main glycosaminoglycan in the mammalian vitreous body, which forms a reticular structure to support collagen fiber scaffold and allows the expansion of gel through swelling osmotic gradient [10, 78]. The human vitreous body is gel-like at birth, but with the growth of age, it will experience an inevitable liquefaction process, and collagen fibers aggregate during age-related vitreous liquefaction [80, 81]. Changes in the composition of ECM and fibrous aggregation appear to be the main events of age-related vitreous liquefaction, thus may result in the weakening of vitreoretinal adhesion and the posterior vitreous detachment, which is implicated in the pathogenesis of many blinding ocular diseases, including rhegmatogenous retinal detachment [77, 78].

## 7. ECM-Based Technology to Treat Ocular Diseases

**Figure 4:**
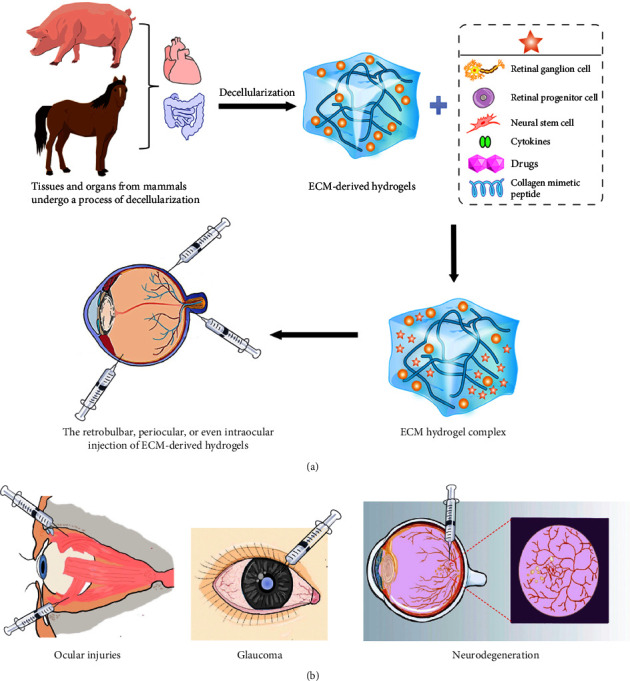
ECM hydrogel for the treatment of ocular lesions. (a) Tissues and organs from different mammals have undergone the decellularized processes to produce ECM-derived hydrogels, which have been developed to combine a variety of components, including RGCs, retinal progenitor cells, NSCs, cytokines, and drugs as well as CMP, shown here with a five-pointed star. Thereafter, ECM hydrogels can be administered as a retrobulbar, periocular, or even intraocular injection for therapeutic purposes. (b) ECM hydrogel for the treatment of ocular injuries, glaucoma, and neurodegeneration. RGCs: retinal ganglion cells. NSCs: neural stem cells. CMP: collagen mimetic peptide.

By 2020, hundreds of millions of people around the world have different degrees of visual impairment as a result of eye diseases and trauma [82]. It is urgent to develop advanced medical strategies to promote beneficial tissue remodeling in the nerve tissue of the eye, retina, and optic nerve. The biological scaffold composed of ECM derived from the decellularized processes of different mammalian tissues or organs has retained many bioactive molecules unique to natural tissues, including collagen, glycosaminoglycans, laminin, and growth factors [83–86] (Figure 4(a)). It has been successfully used in clinical practice to promote the constructive remodeling of many tissues, including skin, heart, esophagus, bladder, and muscle [87, 88]. At present, researchers have gradually applied ECM technology to the field of ophthalmology and developed ECM hydrogel technology and ECM biological hybrid scaffold [87, 89].

### 7.1. ECM Hydrogels

A retrobulbar, periocular, or even intraocular injection can be used to distribute ECM-derived hydrogels since they are organic, biocompatible medical devices with the potential for minimally invasive administration (Figures 4(a) and 4(b)) [87, 90]. In the case of penetrating ocular injuries, local ECM hydrogel injections provide a biocompatible, adjustable gel for bridging acellular injury-induced gaps, enlisting and guiding endogenous stem cell localization and development, and regulate the immune system to accelerate active tissue rebuilding [87, 91]. In the meanwhile, a potential treatment for maintaining visual function is the transplantation of purified retinal progenitor cells or neural stem cells (NSCs) based on the ECM hydrogel platform [87, 92, 93]. Injecting RGCs, progenitor cells, or stem cells intravitreally or into the optic nerve is minimally invasive and has demonstrated neuroprotective benefits in various models of neurodegenerative illness (Figures 4(a) and 4(b)) [94, 95]. Transplanted RGC or stem cells are supposed to retard the onset of retinal degeneration by concurrently controlling a number of pro-survival mechanisms via locally produced neurotrophic factors and/or intraocular microenvironment regulation (Figures 4(a) and 4(b)) [96, 97].

Interestingly, the ocular delivery of collagen mimetic peptide (CMP) compensates for the damage caused by the biochemical degradation and remodeling of ocular ECMs, preventing the functional defect of RGC projected to the brain after the increase of IOP in the most prevalent model of experimental glaucoma, microbead occlusion (Figures 4(a) and 4(b)) [98, 99]. This suggests that intravitreal injection of CMP incorporating the tissue-specific advantages of the ECM hydrogel platform may affect the extensive repair of ECM and promote RGC survival, providing an alternative for patients with chronic and acute optic neuropathy [87, 98].

### 7.2. ECM Biological Hybrid Scaffold

Combining the tunable mechanical and biochemical properties of an electrospun polymer sheet with ECM or ECM-derived meterials, endowing the bio-hybrid scaffold the advantages of higher tensile strength and controllable degradation rates [87, 100]. Researchers have used electrospinning to generate a scaffold with aligned fibers that directionally guide the growth of retinal ganglion axons, which is beneficial for the treatment of glaucoma and other optic neuropathies through cell transplant therapies [101]. Biohybrid scaffolds, which incorporate ECM hydrogels, have stronger biocompatibility than solely synthetic scaffolds and may offer tissue-specific benefits for improving neuropathies and contributing to functional recovery after retinal diseases [87].

## 8. Conclusion

ECM provides attachment and adhesion for cells, regulates intercellular communication, and supports the integrity of multicellular structures. ECM is highly tissue-specific, and its components vary with multicellular structure, affecting a variety of cell life processes. ECM stiffness is a form of mechanical force, and mechanical mechanics is an essential factor in the modulation of cell function and behavior. However, the role of ECM stiffness is rarely discussed in the field of ophthalmology. In this paper, we focus on describing the important role of ECM stiffness and its composition in multiple ocular structures (including cornea, retina, optic nerve, trabecular meshwork, and vitreous body) from a new perspective. Abnormal changes in ECM stiffness and its composition can trigger physiological and pathological activities of the eye in response. This also suggests that, compared with different biochemical factors, ECM stiffness, a mechanical cue, is universal in the transmission and transduction of force signals in different ocular cell types. In addition, we summarized the advantages of using ECM technology as regenerative medicine in the treatment of ocular diseases and emphasized that ECM has great research value and clinical application prospects in the field of ophthalmology.

## Data Availability

The data analyzed in this study is included in this manuscript, and the references supporting the findings of this study are included witin this article.
